# Prognostic role of lymphocyte to monocyte ratio for patients with cancer: evidence from a systematic review and meta-analysis

**DOI:** 10.18632/oncotarget.7876

**Published:** 2016-03-03

**Authors:** Liangyou Gu, Hongzhao Li, Luyao Chen, Xin Ma, Xintao Li, Yu Gao, Yu Zhang, Yongpeng Xie, Xu Zhang

**Affiliations:** ^1^ Department of Urology/State Key Laboratory of Kidney Diseases, Chinese PLA General Hospital/PLA Medical School, Beijing, China; ^2^ School of Medicine, Nankai University, Tianjin, China

**Keywords:** inflammation, lymphocyte to monocyte ratio, cancer, prognosis, meta-analysis

## Abstract

Inflammation influences cancer development and progression, and a low lymphocyte to monocyte ratio (LMR) has been reported to be a poor prognostic indicator in several malignancies. Here we quantify the prognostic impact of this biomarker and assess its consistency in various cancers. Eligible studies were retrieved from PubMed, Embase and Web of Science databases. Overall survival (OS) was the primary outcome, cancer-specific survival (CSS), disease-free survival (DFS), recurrence-free survival (RFS), and progression-free survival (PFS) were secondary outcomes. Pooled hazard ratios (HRs), odds ratios (ORs), and 95% confidence intervals (CIs) were calculated. Fifty-six studies comprising 20,248 patients were included in the analysis. Overall, decreased LMR was significantly associated with shorter OS in non-hematological malignancy (HR: 0.59, 95% CI: 0.53–0.66; *P* < 0.001) and hematological malignancy (HR: 0.44, 95% CI: 0.34–0.56; *P* < 0.001). Similar results were found in CSS, DFS, RFS and PFS. Moreover, low LMR was significantly associated with some clinicopathological characteristics that are indicative of poor prognosis and disease aggressiveness. By these results, we conclude that a decreased LMR implied poor prognosis in patients with cancer and could serve as a readily available and inexpensive biomarker for clinical decision.

## INTRODUCTION

Inflammatory responses play crucial roles at different stages of cancer development and progression and may be linked with systemic inflammation [1–3]. There is increasing evidence that systemic inflammatory response is a key determinant of outcome in patients with cancer, which is reflected by many biochemical or hematological parameters, such as increased C-reactive protein (CRP) levels, hypoalbuminemia or elevated white cell, neutrophil and platelet counts [4]. Several of these parameters have been converted to ratios or prognostic scores such as the neutrophil to lymphocyte ratio (NLR) [5] or the Glasgow Prognostic Score (GPS, combination of CRP and albumin) [6].

Recently, a decreased ratio of peripheral lymphocyte to monocyte ratio (LMR) has been identified as a poor prognostic indicator in various cancers [7–12], which might be a readily available and inexpensive objective prognostic index that could be used to precisely guide clinical decisions. The LMR might be a good reflection of cancer, lymphopenia that is a surrogate marker of weak immune response and an elevated monocyte count, standing for a microenvironment surrogate marker of high tumor burden. However, the consistency and magnitude of the prognostic impact of LMR remain unclear. Therefore, we performed a systematic review of published studies in order to evaluate the prognostic value of LMR by exploring the associations of LMR with survival and the clinicopathological features of cancer. A meta-analysis was conducted with extracted data which could be merged.

## RESULTS

### Included studies

**Figure 1:**
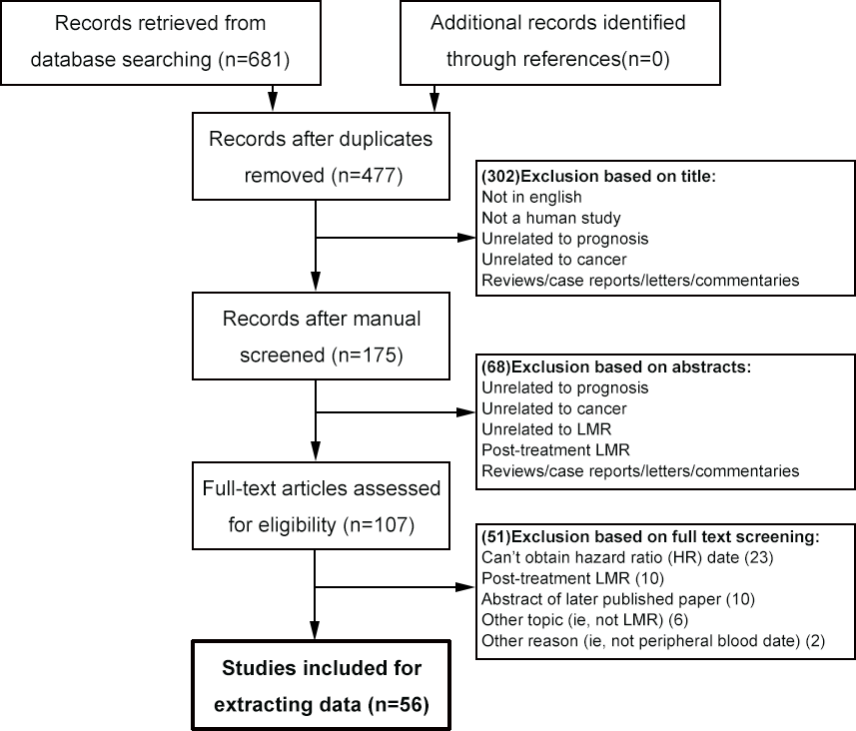
Flowchart of selecting studies for inclusion in this meta-analysis LMR = lymphocyte to monocyte ratio.

The flow chart of the literature search is shown in Figure 1. A total of 681 records were retrieved from a primary literature search in the above databases and no record from references searching, and excluded 204 duplicates from the initial records. After screening the title of 681 studies returned from the search algorithm, 175 studies were selected for reviewing the abstracts. The titles and abstracts screening process identified 107 articles, which met the inclusion criteria. The remaining articles were reviewed in full-text. Finally, 56 articles were included in this meta-analysis.

These studies included a total of 20,248 patients and characteristics of the studies are shown in Table 1 [7–62]. All of the studies were published in 2011 or later. Twenty different types of malignance were analyzed, most of which were lymphoma. Of the 56 identified studies, forty-five were full-text paper and eleven were conference abstract.

**Table 1:** Features of included studies

Features	Studies (*n* = 56)	Patients (*n* = 20248)	References
Year of publication, No. (%)			
2011–2012	7 (12.5)	2499 (12.3)	(59–65)
2013	8 (14.3)	4471 (22.1)	(51–58)
2014	21 (37.5)	7216 (35.6)	(11–12, 32–50)
2015	20 (35.7)	6062 (29.9)	(7–10, 16–31)
Type of publication, No. (%)			
Full paper	45 (80.4)	16511 (81.5)	(7–12, 16–38, 40–42, 44–49, 52, 54, 58, 61–64)
Abstract	11 (19.6)	3737 (18.5)	(39, 43, 50–51, 53, 55–57, 59–60, 65)
Study design, No. (%)			
Prospective	3 (5.4)	801 (4.0)	(8, 22, 34)
Retrospective	53 (64.6)	19447 (96.0)	(7, 9–12, 16–21, 23–33, 35–65)
Type of cancer, No. (%)			
Diffuse large B-cell lymphoma	12 (25.5)	4383 (25.2)	(33–34, 36, 38, 42, 45, 50–51, 54, 60, 63, 65)
Hodgkin's lymphoma	7 (12.5)	2799 (13.8)	(46, 53, 55, 57, 61–62, 64)
Colorectal carcinoma	6 (10.7)	1340 (6.6)	(8, 12, 17, 21–23)
Lung cancer	4 (7.1)	2085 (10.3)	(19, 40, 48–49)
Multiple sites	3 (5.4)	469 (2.3)	(31, 43, 56)
Urothelial carcinoma	3 (5.4)	374 (1.8)	(10, 16, 35)
Renal cell carcinoma	3 (5.4)	1549 (7.7)	(9, 30, 47)
Nasopharyngeal carcinoma	3 (5.4)	2475 (12.2)	(24, 41, 58)
Pancreatic cancer	2 (3.4)	795 (3.9)	(7, 20)
Esophageal carcinoma	2 (3.4)	566 (2.8)	(25–26)
Gastric cancer	2 (3.4)	815 (4.0)	(27, 32)
Burkitt lymphoma	1 (1.2)	62 (0. 3)	(18)
Endometrial cancer	1 (1.2)	605 (3.0)	(28)
Cervical cancer	1 (1.2)	485 (2.4)	(29)
Soft tissue sarcoma	1 (1.2)	340 (1.7)	(11)
Breast cancer	1 (1.2)	542 (2.7)	(37)
Hepatocellular carcinoma	1 (1.2)	210 (1.0)	(39)
Multiple myeloma	1 (1.2)	189 (0.9)	(52)
Melanoma	1 (1.2)	66 (0.3)	(59)
Follicular lymphoma	1 (1.2)	99 (0.5)	(44)
Cancer stage, No. (%)			
Mixed	37 (66.1)	11583 (57.2)	(7, 11, 16, 18–20, 25, 27–28, 30–31, 33–36, 38–39, 42–46, 49–57, 60–65)
Non-metastatic	12 (21.4)	6742 (33.3)	(9–10, 12, 17, 23, 26, 29, 32, 37, 47–48, 58)
Metastatic	7 (12.5)	1923 (9.5)	(8, 21–22, 24, 40–41, 59)
Cutoff for LMR, No. (%)			
1.0 to < 2.0	8 (14.3)	2607 (12.9)	(43, 50, 53, 55–57, 62, 65)
2.0 to < 3.0	25 (44.6)	7978 (39.4)	(7, 9–12, 16, 18, 22–26, 29, 31, 33, 35, 38, 45–46, 52, 54, 59–61, 63–64)
3.0 to < 4.0	9 (16.1)	3517 (17.4)	(8, 17, 19–21, 39, 42, 47–48)
≥ 4.0	13 (23.2)	5703 (28.2)	(27–28, 30, 32, 34, 37, 40–41, 44, 49, 51, 58)
Not reported	1 (1.8)	443 (2.2)	(36)
ROC curve, No. (%)			
Considered	44 (78.6)	17497 (86.4)	(7–12, 18–19, 21–22, 24–29, 32–35, 37–42, 44–54, 58, 60–65)
Not considered	12 (21.4)	2751 (13.6)	(16–17, 20, 23, 30–31, 36, 43, 55–57, 59)
Reported outcome, No. (%)			
Overall survival	44 (78.7)	14984 (72.0)	(8, 10–12, 16, 18–20, 22–24, 26–30, 32–36, 38–43, 45–49, 51–52, 54, 56–59, 61–65)
Cancer-specific survival	11 (18.4)	3972 (18.4)	(7–8, 11, 21–22, 25, 27–28, 47, 61–62)
Recurrence-free survival	7 (12.2)	1849 (7.7)	(12, 29, 32, 35, 38–39, 59)
Progression-free survival	18 (34.0)	5805 (32.6)	(18–19, 31, 34, 39, 42, 44–45, 49, 51, 53,^55^, ^57^, ^60^–^63^, ^65^)
Disease-free survival	15 (26.5)	6440 (34.2)	(8–9, 11, 17, 21, 23, 26–27, 31, 33, 37, 41, 46, 48, 50)

Because of rounding, not all percentages total 100. LMR = lymphocyte to monocyte ratio.

### The prognostic significance of LMR in OS of various cancers

**Figure 2:**
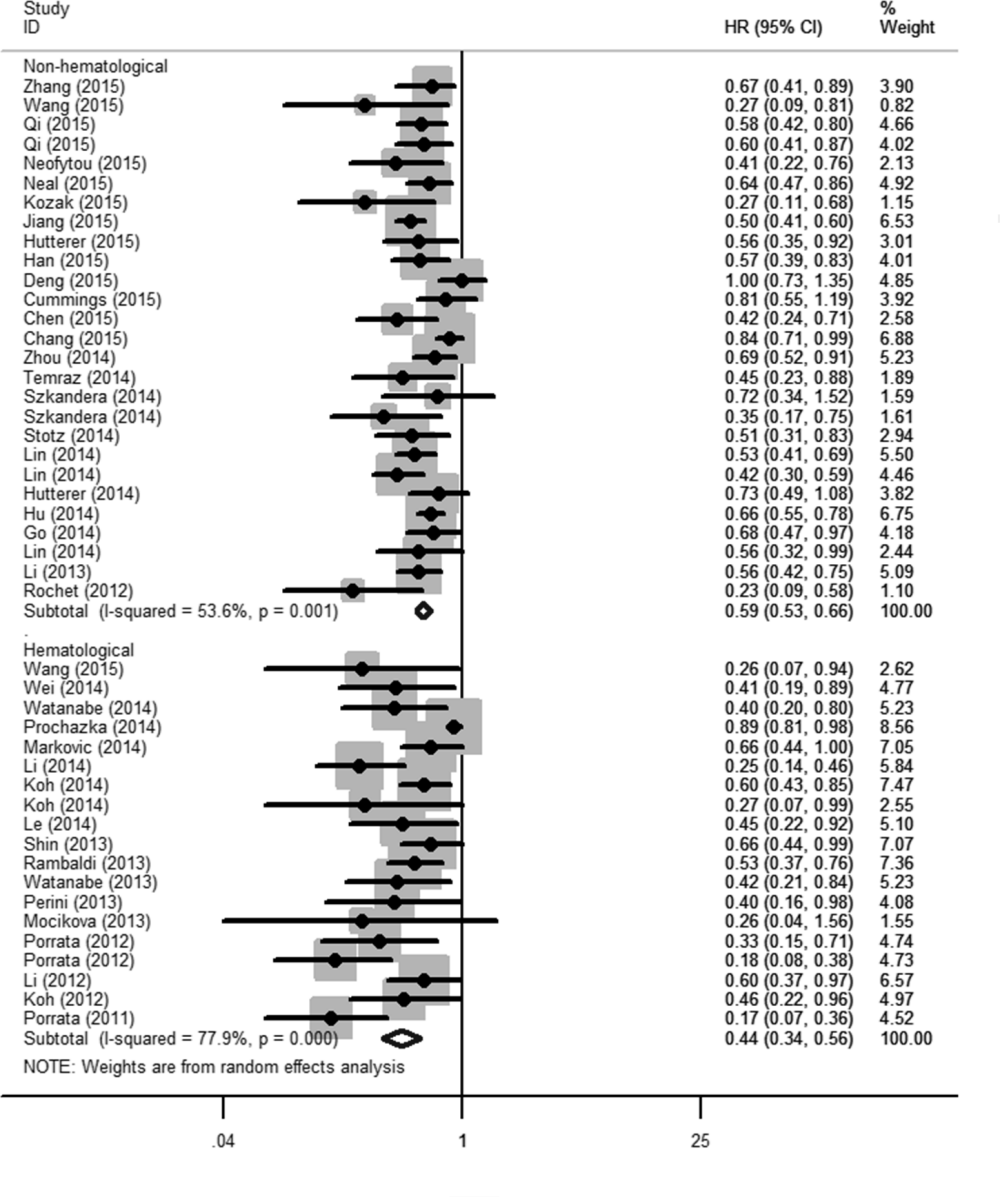
The prognostic significance of lymphocyte to monocyte ratio (LMR) in overall survival (OS) A combined analysis showed that LMR lower than the cutoff was associated with poor OS in non-hematological malignancy (HR: 0.59, 95% CI: 0.53–0.66; *P* < 0.001) and hematological malignancy (HR: 0.44, 95% CI: 0.34–0.56; *P* < 0.001) with significant heterogeneity (*I*^2^ = 53.6% and 77.9%, respectively).

**Figure 3:**
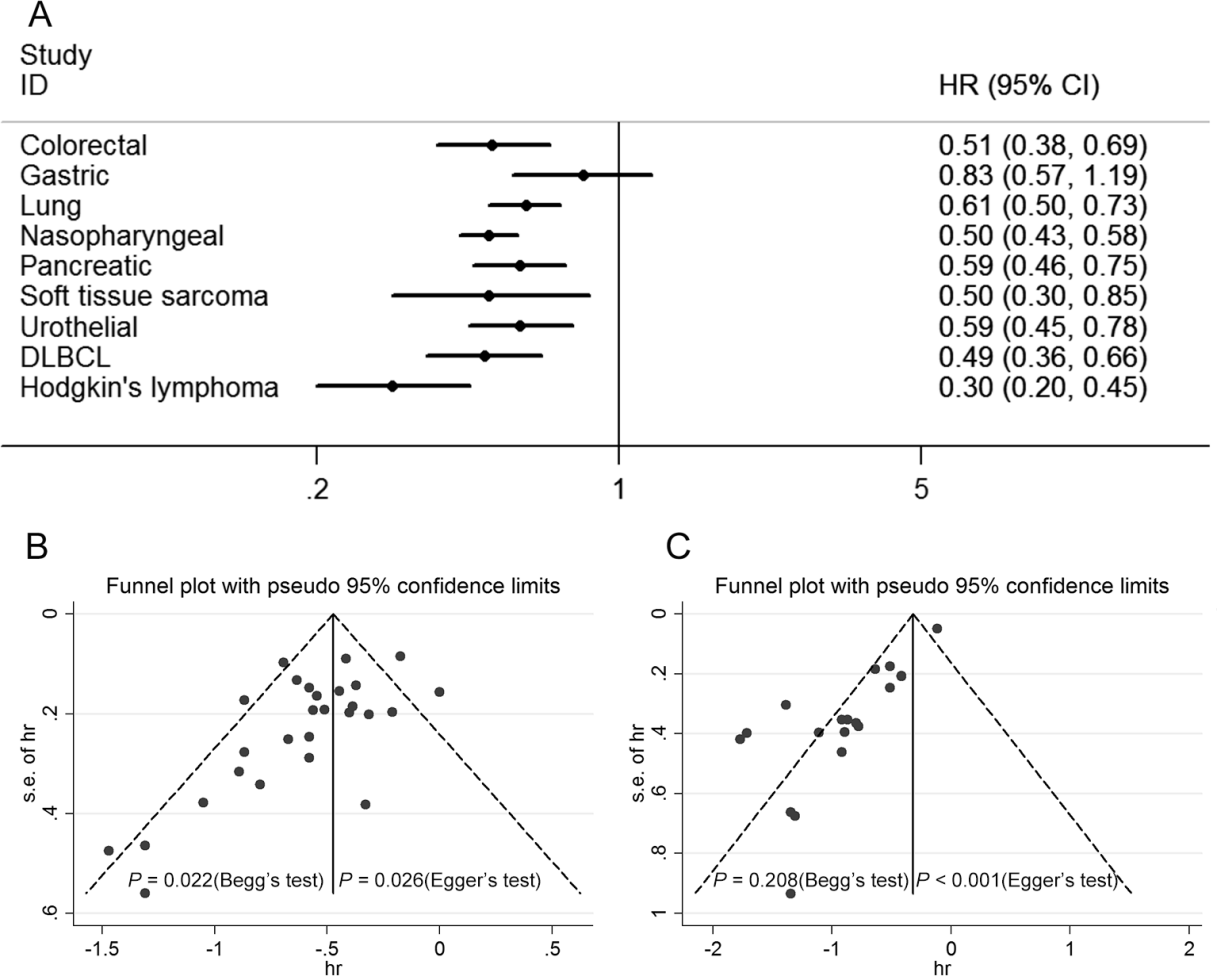
Subgroup analysis of OS by type of cancer and results for the evaluation of publication bias (**A**) The lower LMR was significantly associated with poor OS in colorectal carcinoma, lung cancer, nasopharyngeal carcinoma, pancreatic cancer, soft tissue sarcoma, urothelial carcinoma, DLBCL, Hodgkin's lymphoma but not in gastric cancer. (**B**) The funnel plot for OS of non-hematological malignancy is asymmetric. A publication bias was identified based on Begg's (*P* = 0.022) and Egger's (*P* = 0.026) tests. (**C**) The funnel plot for OS of hematological malignancy is asymmetric. A publication bias was identified based on Begg's (*P* = 0.208) and Egger's (*P* < 0.001) tests.

The association between LMR and OS was reported in 44 studies enrolling 14,984 patients with various cancer types [8, 10–13, 15–17, 19–21, 23–27, 29–33, 35–40, 42–46, 48, 49, 51, 53–56, 58–62]. Five of the eligible 44 studies (11%) reported a non-statistically significant hazard ratio. A forest plot of non-hematological and hematological malignancy is shown in Figure 2. A combined analysis showed that LMR lower than the cutoff was associated with poor OS in non-hematological malignancy (HR: 0.59, 95% CI: 0.53–0.66; *P* < 0.001) and hematological malignancy (HR: 0.44, 95% CI: 0.34–0.56; *P* < 0.001) with significant heterogeneity (*I*^2^ = 53.6% and 77.9%, respectively). The effect of LMR on OS among cancer subgroups is presented in Figure 3A. The lower LMR was significantly associated with poor OS in colorectal carcinoma (HR: 0.51, 95% CI: 0.38–0.69; *P* < 0.001), lung cancer (HR: 0.61, 95% CI: 0.50–0.73; *P* < 0.001), nasopharyngeal carcinoma (HR: 0.50, 95% CI: 0.43–0.58; *P* < 0.001), pancreatic cancer (HR: 0.59, 95% CI: 0.46–0.75; *P* < 0.001), soft tissue sarcoma (HR: 0.50, 95% CI: 0.30–0.85; *P* = 0.01), urothelial carcinoma (HR: 0.59, 95% CI: 0.45–0.78; *P* = 0.001), DLBCL (HR: 0.49, 95% CI: 0.36–0.66; *P* < 0.001), Hodgkin's lymphoma (HR: 0.30, 95% CI: 0.20–0.45; *P* < 0.001) but not in gastric cancer (HR: 0.83, 95% CI: 0.57–1.19; *P* = 0.302). In non-hematological malignancy, subgroup analysis revealed the hazard ratios of LMR on OS among different disease stages were 0.73 (95% CI: 0.66–0.81; *P* < 0.001) for a mixed group comprising studies that included both metastatic and non-metastatic patients, 0.61 (95% CI: 0.55–0.68; *P* < 0.001) for non-metastatic cancer, and 0.50 (95% CI: 0.45–0.57; *P* < 0.001) for metastatic cancer (Table 2).

**Table 2:** Meta-regression and subgroup analysis of LMR and OS of various cancers

Subgroup	HR (95% CI)	*P* value	Meta-regression *P* value	Heterogeneity	
				*I*^2^ (%)	*P* value
Non-hematological					
Year of publication			0.276		
2012–2013	0.40 (0.17–0.93)	0.034		68.7	0.074
2014	0.59 (0.52–0.67)	< 0.001		17.3	0.279
2015	0.61 (0.52–0.72)	< 0.001		65.2	< 0.001
Type of publication			0.250		
Full paper	0.60 (0.54–0.67)	< 0.001		53.3	0.001
Abstract	0.39 (0.16–0.92)	0.031		61	0.109
Study design			0.782		
Prospective	0.56 (0.37–0.83)	0.005		37.6	0.206
Retrospective	0.59 (0.53–0.66)	< 0.001		55.7	< 0.001
Cancer site			0.545		
Cancer stage			0.004		
Mixed	0.73 (0.66–0.81)	< 0.001		45.4	0.043
Non-metastatic	0.61 (0.55–0.68)	< 0.001		3.5	0.405
Metastatic	0.50 (0.45–0.57)	< 0.001		26.7	0.235
Cutoff for LMR			0.015		
2.0 to < 3.0	0.51 (0.45–0.58)	< 0.001		0	0.459
3.0 to < 4.0	0.62 (0.55–0.71)	< 0.001		0	0.497
≥ 4.0	0.67 (0.56–0.80)	< 0.001		69	0.001
ROC curve			0.646		
Considered	0.59 (0.53–0.66)	< 0.001		41.5	0.025
Not considered	0.58 (0.44–0.78)	< 0.001		69.2	0.006
Analysis of hazard ratio			0.950		
Multivariable	0.59 (0.53–0.66)	< 0.001		58.2	< 0.001
Univariate	0.60 (0.48–0.75)	0.001		0	0.644
Hematological					
Year of publication			0.181		
2011–2012	0.32 (0.19–0.55)	< 0.001		63.8	0.026
2013	0.54 (0.42–0.69)	< 0.001		0	0.647
2014–2015	0.48 (0.34–0.69)	< 0.001		78.8	< 0.001
Type of publication			0.207		
Full paper	0.47 (0.36–0.62)	< 0.001		78.9	< 0.001
Abstract	0.34 (0.24–0.50)	< 0.001		0	0.419
Cancer site			0.596		
Cutoff for LMR			0.343		
1.0 to < 2.0	0.27 (0.19–0.40)	< 0.001		19.1	0.293
2.0 to < 3.0	0.56 (0.47–0.65)	< 0.001		0	0.662
≥ 3.0	0.34 (0.23–0.49)	< 0.001		0	0.452
ROC curve			0.203		
Considered	0.43 (0.35–0.54)	< 0.001		48.9	0.017
Not considered	0.57 (0.32–1.01)	0.053		62.4	0.047
Analysis of hazard ratio			0.203		
Multivariable	0.43 (0.35–0.54)	< 0.001		48.9	0.017
Univariate	0.57 (0.32–1.01)	0.053		62.4	0.047

LMR, lymphocyte to monocyte ratio; OS, overall survival; HR, hazard ratio; CI, confidence interval.

Sensitivity analysis indicated that omitting any single study did not significantly affect the pooled HR. In non-hematological malignancy, meta-regression analysis revealed that cancer stage (*P* = 0.004) and cutoff for LMR (*P* = 0.015) might be significant contributors to heterogeneity, whereas publication year, publication type, study design, type of cancer, ROC curve and analysis of hazard ratio were not (*P* = 0.250–0.950). In hematological malignancy, meta-regression analysis revealed that publication year, publication type, cancer site, cutoff, ROC curve and analysis of hazard ratio were not significant contributors to heterogeneity (*P* = 0.181–0.596) (Table 2).

A key point was that the cutoff value varied and ranged from 1.10 to 5.26, which was attributed to the use of different methods and patients’ baseline characteristics (race, country, gender, age, etc.). Moreover, there was a significant association between LMR cutoff and the hazard ratio for OS (*r* = 0.511, *P* < 0.001) (supporting information Figure S1). There was evidence of publication bias in the meta-analysis of the association between LMR and OS, with fewer small studies reporting negative results than would be expected (Figure 3B–3C).

### The prognostic significance of LMR in CSS, DFS, RFS and PFS of cancer patients

**Figure 4:**
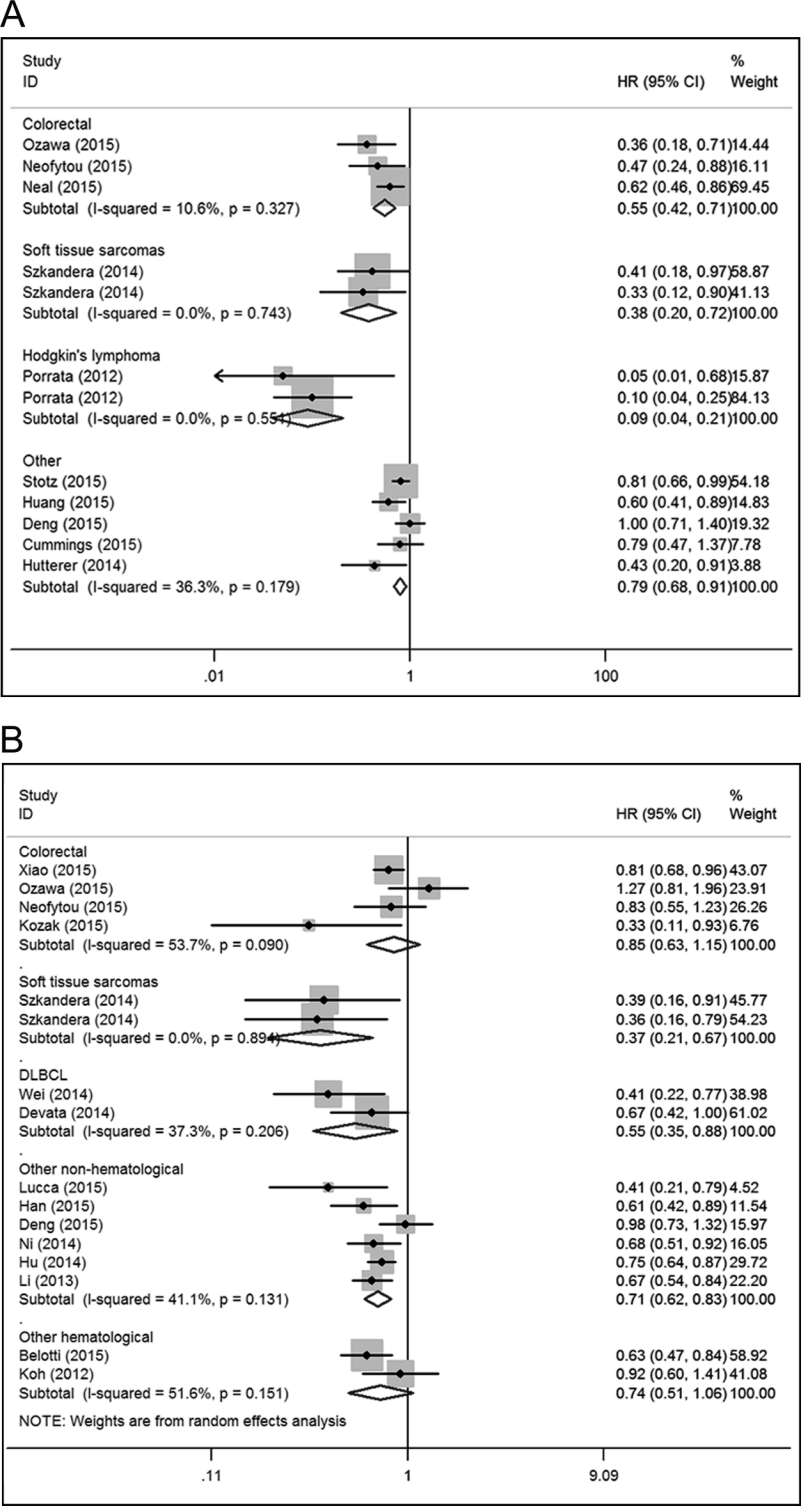
Forest plots for the meta-analysis of the association between LMR and cancer-specific survival (CSS), disease-free survival (DFS) in various cancer types (**A**) The lower LMR was significantly associated with poor CSS in colorectal carcinoma, soft tissue sarcoma, Hodgkin's lymphoma and other non-hematological malignancies. (**B**) The lower LMR was significantly associated with poor DFS in soft tissue sarcoma, DLBCL, other non-hematological malignancies but not in colorectal carcinoma and other hematological malignancies.

**Figure 5:**
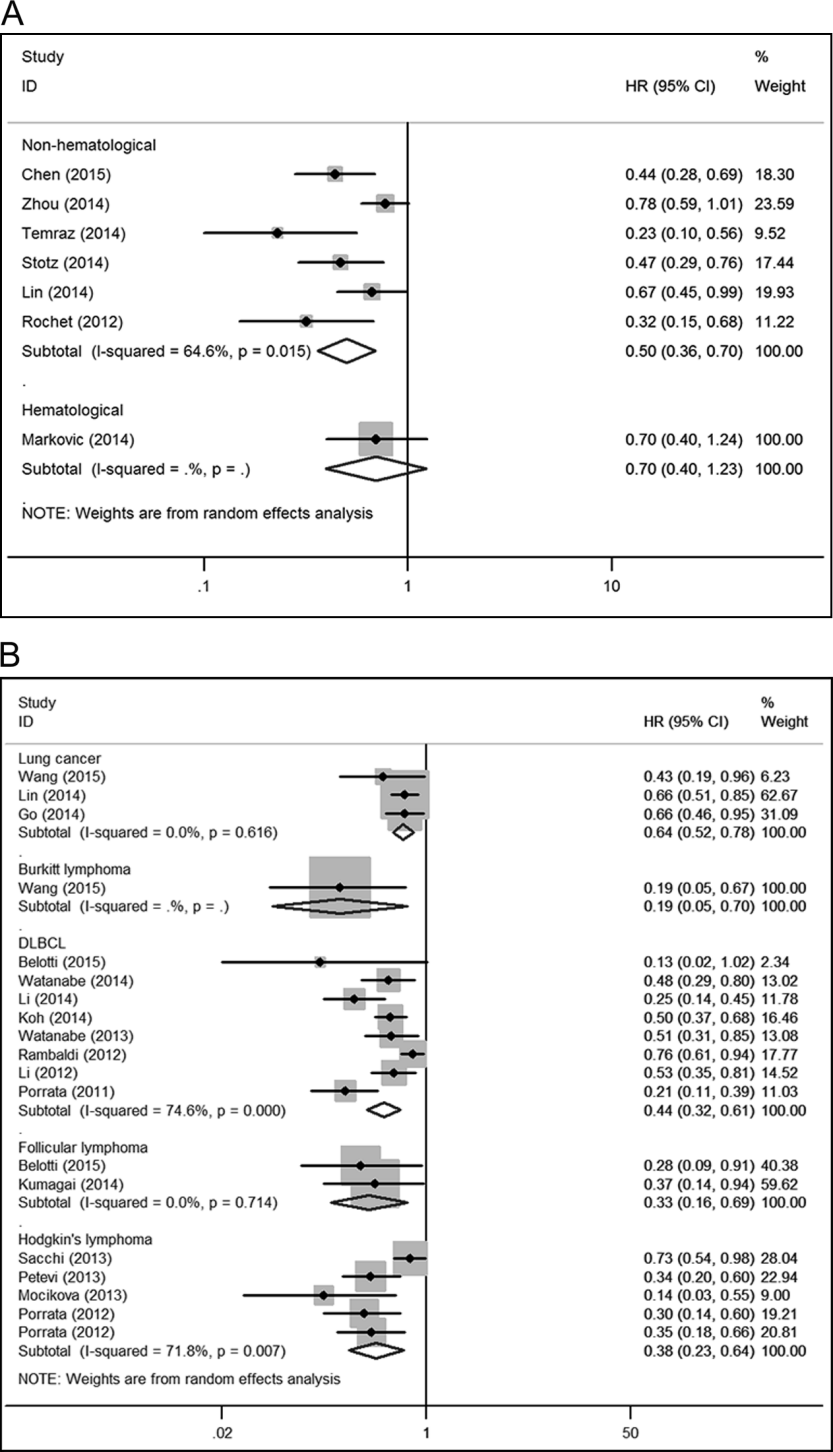
Forest plots for the meta-analysis of the association between LMR and recurrence-free survival (RFS), progression-free survival (PFS) in various cancer types (**A**) A combined analysis showed that LMR lower than the cutoff was associated with poor RFS in non-hematological malignancy. In addition, a non-significant result for DLBCL was reported. (**B**) The lower LMR was significantly associated with poor PFS in lung cancer, DLBCL, follicular lymphoma and Hodgkin's lymphoma. In addition, a significant result for Burkitt lymphoma was reported.

Eleven studies comprising 3,972 patients reported hazard ratios for CSS [7, 8, 11, 18, 19, 22, 24, 25, 44, 58, 59]. The effect of LMR on CSS among cancer subgroups is presented in Figure 4A. The lower LMR was significantly associated with poor CSS in colorectal carcinoma (HR: 0.55, 95% CI: 0.42–0.71; *P* < 0.001), soft tissue sarcoma (HR: 0.38, 95% CI: 0.20–0.72; *P* = 0.003), Hodgkin's lymphoma (HR: 0.09, 95% CI: 0.04–0.21; *P* < 0.001) and other non-hematological malignancies (HR: 0.79, 95% CI: 0.68–0.91; *P* = 0.002). Fifteen studies comprising 6,440 patients reported hazard ratios for DFS [8, 9, 11, 14, 18, 20, 23, 24, 28, 30, 34, 38, 43, 45, 47]. The effect of LMR on DFS among cancer subgroups is presented in Figure 4B. The lower LMR was significantly associated with poor DFS in soft tissue sarcoma (HR: 0.37, 95% CI: 0.21–0.67; *P* = 0.001), DLBCL (HR: 0.55, 95% CI: 0.35–0.88; *P* = 0.013), other non-hematological malignancies (HR: 0.71, 95% CI: 0.62–0.83; *P* < 0.001). However, the association was not significant in colorectal carcinoma (HR: 0.85, 95% CI: 0.63–1.15; *P* = 0.301) and other hematological malignancies (HR: 0.74, 95% CI: 0.51–1.06; *P* = 0.100). Seven studies comprising 1,849 patients reported hazard ratios for RFS [12, 26, 29, 32, 35, 36, 56]. A combined analysis showed that LMR lower than the cutoff was associated with poor RFS in non-hematological malignancy (HR: 0.50, 95% CI: 0.36–0.70; *P* < 0.001). In addition, Markovic et al. [35] reported a non-significant result for DLBCL (HR: 0.70, 95% CI: 0.40–1.23; *P* = 0.212) (Figure 5A). Eighteen studies comprising 5,805 patients reported hazard ratios for PFS [15, 16, 28, 31, 36, 39, 41, 42, 46, 48, 50, 52, 54, 57–60, 62]. The effect of LMR on PFS among cancer subgroups is presented in Figure 5B. The lower LMR was significantly associated with poor PFS in lung cancer (HR: 0.64, 95% CI: 0.52–0.78; *P* < 0.001), DLBCL (HR: 0.44, 95% CI: 0.32–0.61; *P* < 0.001), follicular lymphoma (HR: 0.33, 95% CI: 0.16–0.69; *P* = 0.003) and Hodgkin's lymphoma (HR: 0.38, 95% CI: 0.23–0.64; *P* < 0.001). In addition, Wang et al. [15] reported a significant result for Burkitt lymphoma (HR: 0.19, 95% CI: 0.05–0.70; *P* = 0.012).

### The association between LMR and characteristics of cancer patients

Twenty-one studies provided sufficient data for the meta-analysis of the correlation between LMR and clinicopathological characteristics (Table 3) [10, 22–24, 29–32, 37, 39, 42, 43, 46, 51, 58–61]. Urothelial carcinoma, esophageal cancer, gastric cancer, renal cell carcinoma, lung cancer, DLBCL and Hodgkin's lymphoma were investigated in detail. For each disease excluding urothelial carcinoma, low LMR was significantly associated with some clinicopathological characteristics that are indicative of poor prognosis and disease aggressiveness.

**Table 3:** Results of meta-analysis of LMR and characteristics of six types of cancer

Characteristics	Studies	Patients	OR (95% CI)	*P* value	Heterogeneity	
					*I*^2^ (%)	*P* value
Urothelial carcinoma						
pT stage	3	374	0.86 (0.51–1.47)	0.588	0	0.414
Tumor grade	3	374	1.07 (0.62–1.85)	0.805	0	0.825
Esophageal cancer						
Tumor length	2	566	0.66 (0.44–0.98)	0.041	0	0.466
pT stage	2	566	0.59 (0.40–0.86)	0.007	0	0.808
Lymph node status	2	566	0.59 (0.41–0.84)	0.004	0	0.630
Gastric cancer						
Tumor grade	2	815	0.88 (0.64–1.22)	0.444	17.1	0.272
TNM stage	2	815	0.52 (0.39–0.70)	< 0.001	92.2	< 0.001
Renal cell carcinoma						
Fuhrman grade	2	1119	0.52 (0.39–0.69)	< 0.001	0	0.576
Tumor necrosis	2	1119	0.57 (0.43–0.75)	< 0.001	45.2	0.177
Lung cancer						
ECOG performance status	2	558	0.59 (0.39–0.90)	0.013	47.1	0.169
Diffuse large B-cell lymphoma						
Ann Arbor stage	6	2869	0.42 (0.36–0.49)	< 0.001	29.3	0.194
IPI score	4	1907	0.38 (0.31–0.47)	< 0.001	0	0.727
ECOG performance status	5	2701	0.39 (0.31–0.48)	< 0.001	74.3	0.001
Extranodal sites of disease	6	2869	0.58 (0.48–0.69)	< 0.001	61.6	0.011
Serum LDH level	6	2869	0.27 (0.23–0.32)	< 0.001	68.9	0.002
B symptom	2	962	0.38 (0.25–0.58)	< 0.001	0	0.364
Hodgkin's lymphoma						
Ann Arbor stage	4	1188	0.42 (0.33–0.53)	< 0.001	78.6	0.003
Stage	3	1085	0.40 (0.31–0.52)	< 0.001	81.5	0.005
IPS	2	609	0.26 (0.16–0.42)	< 0.001	0	0.723
WBC count	4	1188	0.67 (0.46–0.99)	0.047	0	0.686
Albumin	4	1188	0.47 (0.36–0.60)	< 0.001	68	0.025
Hemoglobin	4	1188	0.41 (0.30–0.56)	< 0.001	57.8	0.068

LMR, lymphocyte to monocyte ratio; OR, odds ratio; CI, confidence interval; ECOG, eastern cooperative oncology group; IPI, international prognostic index; LDH, lactate dehydrogenase; IPS, international prognostic score.

Regarding predictive factors for DLBCL, low LMR was significantly associated with high serum LDH level (HR: 0.27, 95% CI: 0.23–0.32; *P* < 0.001). Regarding predictive factors for Hodgkin's lymphoma, low LMR was significantly associated with high white blood cell count (HR: 0.67, 95% CI: 0.46–0.99; *P* = 0.047), low albumin (HR: 0.47, 95% CI: 0.36–0.60; *P* < 0.001) and low Hemoglobin (HR: 0.41, 95% CI: 0.30–0.56; *P* < 0.001).

## DISCUSSION

The post-operative histopathological parameters, which mainly focus on the biological behavior and presentation of the tumor itself, act as the foundation for subdividing cancer patients and determining the suitable treatment. However, these variables might not be entirely reliable for predicting the prognosis precisely and guiding the clinical practice appropriately. The introduction of the laboratory index as a supplementary item to current prognostic prediction system is required for tailoring the personalized treatment strategy.

So far, the prognostic significance of the markers of systematic inflammatory response to solid tumors has been identified [63–65]. A variety of recent studies have suggested that a decreased LMR is associated with poor survival of subjects with cancer. In the present study, we showed that decreased pretreatment LMR has an unfavorable impact on OS in cancer patients among various disease subgroups. Inflammation has been reported to contribute to the development of many tumors and is now considered as a hallmark of cancer [66]. Additionally, we found a trend for the association of low LMR with poor OS to be greater for metastatic than non-metastatic cancer, which may reflect either higher tumor burden or a more prolonged chronic inflammatory process [2]. The prognostic impact of LMR on CSS, DFS, RFS and PFS was retained across cancer sites. A key point of our study was that the cutoff level varied and ranged from 1.1 to 5.26, and although some studies reported that cutoffs were determined using receiver operating characteristic curves (ROC), the approach of choosing LMR cutoffs remained unclear in many papers. And we identified that there was a correlation between LMR cutoff and reported hazard ratio for OS. Koh et al. identified the LMR being related to the age of patients. Therefore it also is imaginable that the optimal cutoff has to be adjusted based on now unknown clinicopathological parameters and/or by each tumor entity on its own [42].

Our results also indicate that LMR was associated with tumor length, pT stage, lymph node status in esophageal cancer. LMR was associated with TNM stage in gastric cancer. LMR was correlated with Fuhrman grade, tumor necrosis in renal cell carcinoma. LMR was correlated with ECOG performance status in lung cancer. Further, we found that LMR was associated with Ann Arbor stage, IPI score, ECOG performance status, extranodal sites of disease, serum LDH level and B symptom in DLBCL, and with Ann Arbor stage, stage, IPS, WBC count, albumin level and hemoglobin level in Hodgkin's lymphoma. As LMR measurement is well standardized and available in every clinical laboratory, it could be a helpful and convenient serum biomarker for clinical practice.

Our results agree with several studies conducted at the same time [67–69]. Compared with these publications, our meta-analysis has several strengths. First, in contrast to these studies focusing on DLBCL or non-hematological solid tumors, we studied all types of malignancy. Because of the different tumor biology, the meta-analyses were performed for non-hematological and hematological malignancy respectively. Second, besides outcomes reported in these studies, we also investigated the RFS and PFS for non-hematological solid tumors, and CSS for hematological malignancy, which provided comprehensive evidence for the prognostic role of LMR in patients with cancer. In addition, the relationships between LMR and the features of tumor patients were also studied in our meta-analysis, which were never discussed in these studies. Hence, to our knowledge, the present meta-analysis is the most comprehensive and informative study.

Significant heterogeneity was observed in most of our analyses. Subgroup analyses were preformed to present more results in detail. Meta-regression and sensitivity analyses did not alter the significant correlation of LMR with survival outcomes and reveal some significant sources of heterogeneity. However, certain stratifying covariates might contribute to the limited statistical power of meta-regression.

The reasons why LMR might be of prognostic relevance in patients suffering cancer remain speculative at this time. Lymphocyte is a key mediator of immunosurveillance and immune-editing, and lymphocyte infiltration into the tumor microenviroment is a prerequisite to an immunologic antitumor reaction [70–72]. The presence of tumor-infiltrating lymphocytes is related to improved survival in diverse cancers, and conversely, low lymphocyte counts and failure to infiltrate the tumor lead to inferior survival [71, 73]. In addition, CD 8+ and CD 4+ T-lymphocyte interaction among each other could be proven to be essential in anti-tumor reaction of the immune system, by inducing tumor cell apoptosis [74, 75]. Generally, a low lymphocyte amount could be the reason for a weak, insufficient immunologic reaction to the tumor [71]. Nevertheless, monocytes infiltrating tumor tissue also have an effect on tumor development and progression [3]. Monocytes exert a major role in innate immunity, constitute about 5% of the circulating white blood cell pool and exhibit a short half-life in the circulation of a few hours [76]. Macrophages, which are more differentiated monocytes, develop from cells of the mononuclear phagocytic lineage and show specific phenotypic characteristics. The role of macrophages/monocytes in cancer development and progression is disputed, since they have inhibiting as well as enhancing potential of monocytes in human cancer [77]. Nevertheless, there is increasing evidence that the tumor-associated macrophages (TAMs) enhance tumor progression. Bingle et al. [78] showed poor clinical outcome associated with macrophage density in various tumor entities. Pollard and Condeelis et al. [79, 80] found that macrophages support tumor cell migration, invasion and intravasation as well as tumor-associated angiogenesis and even lead to a suppression of anti-tumor immune reaction. A major lineage regulator for macrophages, colony-stimulating factor 1 (CSF-1) [81], was shown to be associated with poorer prognosis in different cancer types [82]. Evani et al. [83] showed that monocytes play a role in metastasis of breast cancer by mediating the adhesion of tumor cells to the endothelium. Condeelis and Pollard found similar results, implicating macrophages for tumor cell migration and invasion [79]. Moreover, primary inflammatory macrophages change in tumor from phenotype to macrophages similar to those that play a role in the regulation of tissue formation during development [81]. Additionally, Lin et al. [84] and Jetten et al. [85] gave insight into the role of macrophages in angiogenesis and vascular remodeling induced by them in tumor formations. All this data suggests a pro tumorous potential of monocytes due to formation of diverse macrophage phenotypes that facilitate the malignant process.

Several limitations of this study need to be acknowledged. Only summarized data rather than individual subject data could be used. Second, we found evidence of publication bias, with fewer small studies reporting negative results than would be expected (Figure 3B–3C), which cannot be properly overcome by statistical techniques. Moreover, marked heterogeneity of subjects was seen in most of analyses. Meta-regression analysis revealed that cancer stage and cutoff for LMR might be significant contributors to heterogeneity for OS in non-hematological solid tumors. Also, the heterogeneity of the population was probably due to differences in factors such as study design, assay methods, patients’ baseline characteristics (race, country, gender, age, and tumor stage and grade), patients’ treatment, and duration of follow-up. In addition, the source of hazard ratio and method of calculating the HRs of these studies was also a potential factor that might have led to heterogeneity. Of the 56 studies, 50 directly provided HRs, and individual HRs of the remaining studies were calculated using the methods reported by Tierney et al. [86]. The calculated HRs could be not as dependable as those retrieved directly from reported statistics. Among the 50 studies providing HRs, five reported univariate hazard ratios, which could introduce a bias toward overestimation of the prognostic role of LMR [8, 18, 19, 33, 54]. In some studies, hazard ratios from multivariable analysis may not have been statistically significant, this might be attribute to inclusion of other markers of systemic inflammation in the multivariable model, which may provide similar information to LMR and thus lead to a non-statistically significant outcome [11, 24, 25]. Furthermore, lymphocyte and monocyte count are nonspecific parameters, which may be influenced by concurrent conditions such as inflammation, infections, and medications. However, most studies reported LMR ahead of surgery or before start of systemic treatment. Despite this, the confounding effect of concurrent inflammatory conditions can't be completely excluded. Finally, the unavoidable limitations exist. All meta-analyses are affected by the quality of their component studies; the fact that research with statistically significant results is potentially more likely to be submitted and published than work with null or non-significant results, could compromise the validity of such analyses. Furthermore, the current meta-analysis of published studies does not have the benefit of currently unpublished data. Owing to further groundbreaking research on inflammation and tumors, we believe that the use of LMR as a prognostic marker for cancer will be extensively studied, and additional studies supporting our results will facilitate a consensus on this matter.

Our comprehensive meta-analysis strongly supports a low LMR is associated with adverse survival in various cancers. The relative availability and low cost of this biomarker should facilitate its use in this context, although a large prospective study is needed to confirm our findings.

## MATERIALS AND METHODS

This meta-analysis was conducted following the guidelines of the Meta-analysis of Observational Studies in Epidemiology group (MOOSE) [87] and Preferred Reporting Items for Systematic Reviews and Meta-analysis (PRISMA) criteria [88].

### Search strategy

A systematic literature search was performed using PubMed, Embase and Web of Science databases. Our search strategy included terms for: “LMR” (e.g., “lymphocyte to monocyte ratio,” “lymphocyte monocyte ratio,” “lymphocyte-to-monocyte ratio”), “prognosis” (e.g., “prognosis,” “outcome,” “survival,” “mortality,” “recurrence” “progression,” “metastasis”) and “cancer” (e.g., “cancer,” “tumor,” “neoplasm” “carcinoma”). The literature search was conducted in July 2015. Additionally, we manually screened the references from the relevant literature, including all of the identified studies, reviews, and editorials.

### Study selection

Inclusion criteria for selecting the articles for our analysis were as follows: (1) studies of people with cancer reporting on the prognostic impact of the peripheral blood LMR; (2) measurement of LMR before specific treatments; (3) clearly described outcome assessment by representing it in overall survival (OS), cancer-specific survival (CSS), disease-free survival (DFS), recurrence-free survival (RFS) or progression-free survival (PFS); (4) survival outcome was further explored considering Hazard ratio (HR) with Confidence interval (CI), HR with *P* value, Kaplan-Meier curves or the required data for calculating HR and CI; (5) retrospective or prospective study design; (6) median follow-up of at least 6 months.

Exclusion criteria were as follows: (1) were not written in English; (2) were letters, editorials, expert opinions, reviews, case reports; (3) non-human research; (4) sampled fewer than 40 patients; (5) dealt with LMR as a continuous rather than a dichotomized variable; or (6) lacked sufficient data for estimating HRs and their 95% CIs. When duplicate studies were retrieved, we included the more informative and recent article. Three reviewers (L.Y.G., H.Z.L. and L.Y.C) identified all the studies that fit the inclusion criteria for full review. Discrepancies were resolved through discussion.

### Data extraction and synthesis

OS was the primary outcome of interest. CSS, DFS, RFS and PFS were secondary outcomes. The three investigators (L.Y.G., H.Z.L. and L.Y.C) extracted data independently, used a predefined form to extract all relevant information. The following details were extracted: the first author's last name, year of publication, type of publication (full text, abstract), study design, number of patients, type of cancer, cancer stage (non-metastatic, metastatic, mixed [non-metastatic and metastatic]), cut-off value, receiver operating characteristic (ROC) curves considered for selection of cut-off, and HR and 95% CI for OS, CSS, DFS, RFS or PFS as applicable. HRs were extracted preferentially from multivariable analyses where available. Otherwise, HRs from univariate analyses were extracted. When HRs were not provided, we calculated them with the original study data (Kaplan-Meier curves or the required data) by using the methods reported by Tierney et al. [86]. For OS, because of the different tumor biology, the meta-analyses were performed initially for all included studies in non-hematological and hematological malignancy respectively. Then, meta-analyses were performed according to different types of malignancy. In order to explore source of heterogeneity, subgroup analyses were also conducted for predefined parameters such as study design, type of publication, type of cancer, cancer stage and so on. Subgroups were generated if at least two studies on that were available. For secondary outcomes, meta-analyses were performed according to different types of malignancy.

The relationships between LMR and the features of tumor patients were also studied. Data on pathological *T* stage (T2-4 versus T1), tumor grade (III/IV versus I/II) of urothelial carcinoma; tumor length (> 3 versus ≤ 3), pathological T stage (T3/T4 versus T1/T2), lymph node status (positive versus negative) of esophageal cancer; tumor grade (III/IV versus I/II), TNM stage (III/IV versus I/II) of gastric cancer; Fuhrman grade (III/IV versus I/II), tumor necrosis (present versus absent) of renal cell carcinoma; ECOG performance status (≥ 2 versus < 2) of lung cancer; Ann Arbor stage (III/IV versus I/II), IPI score (> 2 versus ≤ 2), ECOG performance status (> 1 versus ≤ 1), extranodal sites (≥ 2 versus < 2), serum LDH level (high versus normal), B symptom (present versus absent) of diffuse large B-cell lymphoma (DLBCL); Ann Arbor stage (III/IV versus I/II), stage (III/IV versus I/II), IPS (≥ 4 versus < 4), WBC count (× 10^3^ cells/μ l) (≥ 15 versus < 15), albumin (g/dl) (≥ 4 versus < 4), hemoglobin (g/dl) (≥ 10.5 versus < 10.5) of Hodgkin's lymphoma were dichotomized. The odds ratio (OR) and corresponding 95% CI were extracted and used in meta-analysis.

### Statistical analysis

A test of heterogeneity of combined HRs and ORs was conducted using Cochran's *Q* test and Higgins *I*-squared statistic. A *P* value of less than 0.1 was considered significant. *I* ^2^ > 50% is considered as a measure of severe heterogeneity. When heterogeneity was significant, we used a random-effect model. Otherwise, we used a fixed-effect model. An observed HR or OR < 1 implied poor survival for the group with a low LMR or a significant association between a low LMR and patients features. We pooled HRs and ORs of the studies by using Stata 12.0 software (StatCorp, College Station, TX, USA). The reasons for inter-study heterogeneity were also explored by using meta-regression analysis and subgroup analysis. We also conducted sensitivity analysis by omission of each single study to evaluate stability of the results. The correlation of cutoff and HR for OS was analyzed by linear regression analysis. To assess the risk of publication bias, we used a funnel plot, the Begg's and Egger's test for outcomes when at least 10 studies were included in the meta-analysis. All statistical tests were two-sided, and statistical significance was defined as *P* less than 0.05.

## SUPPLEMENTARY MATERIALS FIGURE


