# Supplementary material for: Prognostic role of lymphocyte to monocyte ratio for patients with cancer: evidence from a systematic review and meta-analysis
Source: Oncotarget. 2016 Mar 3;7(22):31926–42. doi: 10.18632/oncotarget.7876 (PMC5077986; doi:10.18632/oncotarget.7876)
Supplement: Supplementary file 1 [file oncotarget-07-31926-s001.pdf]

Prognostic role of lymphocyte to monocyte ratio for patients with cancer: evidence from a systematic review and meta-analysis

Supplementary Materials

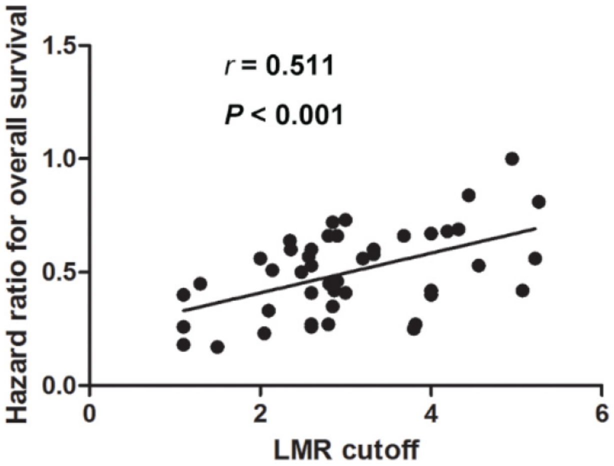

Supplementary Figure S1: Hazard ratio for LMR was correlated with cutoff.
